# Presenting Survey Items One at a Time Compared to All at Once Decreases Missing Data without Sacrificing Validity in Research with Internet Volunteers

**DOI:** 10.1371/journal.pone.0036771

**Published:** 2012-05-17

**Authors:** Brian A. Nosek, N. Sriram, Emily Umansky

**Affiliations:** Department of Psychology, University of Virginia, Charlottesville, Virginia, United States of America; Research and Development Corporation, United States of America

## Abstract

In two large web-based studies, across five distinct criteria, presenting survey items *one-at-a-time* was psychometrically either the same or better than presenting survey items *all-at-once* on a single web page to volunteer participants. In the *one-at-a-time* format, participants were no more likely to drop-out of the study (Criterion 1), and were much more likely to provide answers for the survey items (Criterion 2). Rehabilitating participants who otherwise would not have provided survey responses with the *one-at-a-time* format did not damage internal consistency of the measures (Criterion 3) nor did it negatively affect criterion validity (Criterion 4). Finally, the *one-at-a-time* format was more efficient with participants completing it more quickly than the *all-at-once* format (Criterion 5). In short, the *one-at-a-time* format results in less missing data with a shorter presentation time, and ultimately more power to detect relations among variables.

## Introduction

Survey researchers spend a good deal of time and effort considering what content to measure, but sometimes little effort in how to measure it. However, the quality of collected data can be significantly impacted by the measurement procedures. Maximizing procedural quality impacts the quality of the measured content, and ultimately the power, reliability and validity of the results and conclusions.

Researchers have investigated some differences between survey methodologies in computer-based research. For example, previous investigations compare paging versus scrolling layouts, radio versus drop-down response formats, and the effects of varying amounts of text per screen [1]–[3]. In this article, we report randomized experimental trials comparing two survey presentation formats that are common, especially in web-based research: presenting items *all-at-once* on a single web page, and presenting items *one-at-a-time* on separate pages. In the past decade, researchers have drawn various comparisons between these types of survey methods: some report internal consistency or correlations with criterion variables across the two methods, some compare the length of time the surveys take, and some examine missing or non-substantive data rates [4]–[6]. Moreover, not all research on this comparison uses a strict *one-at-a-time* format; instead, some opt for a mixed approach that occasionally displays several related items on the same screen [1], [7].

We conducted a comprehensive investigation of *one-at-a-time* versus *all-at-once* methods. We evaluated these formats with five distinct evaluation criteria using very large samples and observed that the *one-at-a-time* format performed substantially better across evaluation criteria. The *one-at-a-time* format is likely to provide benefits in increasing the power of survey measurement, especially in the context of participants that do not already have strong incentives to answer survey items (e.g., volunteers).

### All-at-once Survey Format

**Figure 1:**
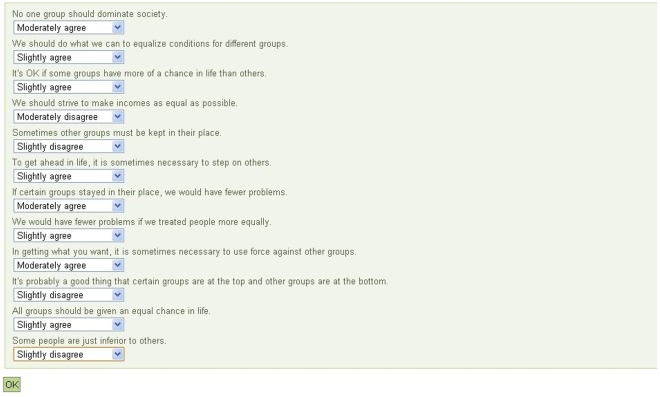
Visual representation of the *all-at-once* format for the Social Dominance Orientation survey.

The *all-at-once* format is designed such that all items in a given questionnaire are displayed on a single screen, with response options available for each after clicking a drop-down menu button (see Figure 1). If the number of response options is long, the drop-down menu presents a subset of response options and the user can scroll to see the remaining options. Participants respond to all of the items and then submit their answers all at once. This format allows participants to see exactly what items remain to be answered. Reaction time data for responses to individual items are not recorded in this format.

### One-at-a-time Format

**Figure 2:**
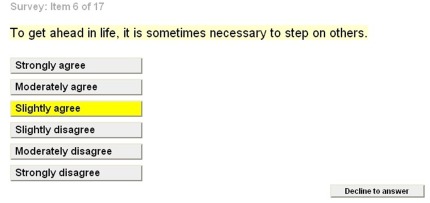
Visual representation of the *one-at-a-time* format for a single item from the Social Dominance Orientation survey.

The *one-at-a-time* format presents a single item on the screen instead of several items at once. On screen are the instructions associated with the item, the item itself, and the response options. All of the response options are displayed on the screen at once (see Figure 2). To select a response, participants click their chosen response once, turning the response button yellow. To confirm the selection, participants click the selected (yellow) response again, thereby answering the item and moving forward to the next item in the survey. Alternatively, for questions allowing multiple responses, respondents click as many items as are relevant once, and then confirm their selection by selecting “next” to move on to the next screen. For each item, participants can select “Decline to Answer” to move on to the next item without providing a response. So, in contrast to the *all-at-once* format, participants must make a response to every single item – if only to say that they decline to answer.

Throughout the questionnaire, participants can view their progress in the survey via a counter near the top of the screen which identifies the question number they are on and the total number of questions for that survey. This format also records the length of time it takes to respond starting from the time the question is displayed.

### Evaluation Criteria

There are multiple criteria for evaluating the better procedure for administering survey items to volunteer participants. Our studies employed volunteers in a web-based study administration. Volunteers in web-based research do not have social normative or compensation factors that motivate them to complete study materials. As such, if measures are boring, frustrating, or confusing, then participants are likely to simply drop-out of the study. Better procedures will minimize participant attrition (criterion 1). In some cases, participants can be required to answer questions before continuing to the next stages of the study. However, this can be annoying if participants do not want to answer a question. Also, in most research applications, participants must have the option to decline to answer for ethical purposes. As such, when participants have the option to decline to answer, better procedures will minimize the likelihood that participants choose not to answer questions (criterion 2). If participants do answer, the researcher hopes that the responses are the the respondent’s best effort to answer the question asked. As such, better procedures will elicit higher internal consistency among items that are supposed to be intercorrelated (criterion 3), and stronger relations between the responses and criterion validity variables (criterion 4). Finally, for both participants and researchers, better procedures will be able to collect quality data in the shortest possible time (criterion 5). Procedures that operate efficiently and quickly allow administration of more items in the same period of time, and respects the fact that respondents probably have other things to do. In two studies, we evaluated *one-at-a-time* and *all-at-once* survey procedures according to these five criteria.

## Experiment 1

Project Implicit (https://implicit.harvard.edu/) is a web-based research laboratory that enjoys heavy traffic averaging more than 20,000 completed study sessions per week from Internet volunteers. The large volume of participants is a function of a persistent media presence and integration into a variety of academic and organizational education practices for learning about implicit cognition – thoughts and feelings outside of conscious awareness or conscious control. Visitors to the “demonstration” side of the website can complete a variety of implicit measures, most notably the Implicit Association Test (IAT) [8] to assess associations that they may possess about people and social groups that they did not know they had, and may even actively disagree with. Most studies offered at the website include an IAT and some brief surveys measuring demographic information and content relevant to the topic of the IAT such as attitudes about various social groups. The studies require 10–15 minutes to complete and participants receive feedback about their IAT performance at the end.

The IAT, and implicit measurement, is not the substantive component of investigation in this article. Rather, we used this large, convenient data collection mechanism to introduce a randomized experimental trial of the two survey formats. Accompanying the skin-tone IAT [9] – a task measuring associations between the social categories *light-skinned people* and *dark-skinned people* and the attributes *good* and *bad* – we presented a popular 12-item measure called Social Dominance Orientation (SDO) [10] plus five additional items measuring attitudes toward light- and dark-skinned people and self-assessed skin-tone in either the *all-at-once* or *one-at-a-time* formats.

### Methods

#### Participants

Visitors to Project Implicit (https://implicit.harvard.edu/) self-selected to participate in the “skin-tone” task from a group of about a dozen studies. 12461 sessions were initiated between March 1, 2010 and April 1, 2010. The participants of 9219 of those sessions remained in the study all the way through the debriefing (74%). Of participants completing demographic information, 72% were female, the average age was 27 (*SD* = 10.4), and 59% were White, 3% East Asian, 3% South Asian, 20% Black, 6% multiracial, and the rest other or unknown.

#### Materials

Social Dominance Orientation (SDO) [10]. SDO measures participants agreement with 12 statements such as “Some people are just inferior to others” and “We would have fewer problems if we treated people more equally” (reverse-coded) on a six-point scale from 1 = strongly disagree to 6 = strongly agree. SDO is a popular measure and relates to attitudes toward social groups [11]–[12]. Typically, participants scoring higher on the SDO report more positive attitudes toward dominant social groups and more negative attitudes toward subordinate social groups than do people scoring lower on the SDO. Also, political conservatives, men, and older adults tend to score higher on the SDO compared to political liberals, women, and younger adults. These known relations served as our criterion validity tests for comparing the survey formats.

##### Skin-tone self-reported attitudes

Three items measures skin-tone attitudes: (1) rating of preference for light-skinned or dark-skinned people on a 7-point scale from strongly prefer light-skinned people to strongly prefer dark-skinned people, (2) rating feelings of warmth for light-skinned people on an 11-point scale from 1 = extremely cold to 11 = extremely warm, (3) rating feelings of warm for dark-skinned people using the same scale, (4) self-rating of one’s own skin-tone from 1 = very dark to 7 very light, and (5) self-rating of one’s preferred personal skin-tone from 1 = be much darker to 5 = be much lighter. These five items were incorporated into the same survey as the SDO making the survey length 17 items in both procedure formats.

##### Implicit Association Test (IAT) [8]

The IAT is a behavioral task in which stimulus items representing two categories (*light-skinned people, dark-skinned people*) and two evaluations (*good, bad*) appear one at a time on the computer screen and must be categorized into their respective superordinate categories as quickly as possible (for a review of psychometric properties and procedures see [13]). There are two critical response blocks in the task: (1) participants categorize *light-skinned faces* and *good words* with one response key, and *dark-skinned faces* and *bad words* with an alternate response key; and (2) participants categorize *dark-skinned faces* and *good words* with one response key, and *light-skinned faces* and *bad words* with the alternate response key. Participants who are able to complete the first categorization task faster than the second are understood to have stronger associations of *light-skinned faces* with *good* and *dark-skinned faces* with *bad* compared to the reverse. The order of the two critical response blocks is counter-balanced across participants, and practice blocks orient participants to the stimuli and task instructions. The procedure followed recommendations by Nosek et al. (2007) [13], and the skin-tone task was the same as reported in Nosek, Smyth, et al. (2007) [9]. Response times for the categorization task were analyzed using the recommended *D* scoring algorithm [14]. Positive values indicate an implicit preference for light-skinned faces compared to dark-skinned faces; negative values indicate an implicit preference for dark-skinned faces compared to light-skinned faces.

##### Demographics

A demographics questionnaire of 13 items also appeared in the study session and was presented in the *all-at-once* format for *all* participants. Two items from the demographics questionnaire are used in the present article: (1) self-reported political orientation on a 7-point scale from 1 = strongly liberal to 7 = strongly conservative; and (2) age in years.

#### Ethics statement

The University of Virginia Institutional Review Board for the Social and Behavioral Sciences approved this research and informed consent process (#2002–0232). Participants were given written informed consent prior to participation, and received a written debriefing at the end of the study session.

#### Procedure

After selecting to complete the skin-tone task, participants initiated a session and encountered an introductory page briefly describing the study procedure and informing participants that they would receive feedback on their task performance at the end. Then, participants completed the SDO, skin-tone IAT, self-report measures, and demographics measures in a randomized order. Self-report measures and demographics were each presented on a single page in the *all-at-once* format. The 12-item SDO +5 additional items were presented in either the *all-at-once* format or with the *one-at-a-time* format selected at random between participants. After completing all measures, participants were given feedback on their IAT performance and debriefed.

### Results

#### Criterion 1 - Attrition

Of the 5237 participants that received the SDO in the *all-at-once* condition, 4510 finished the study (86.1%). Almost the same percentage of the 5500 participants in the *one-at-a-time* condition finished the study (4709, 85.6%). Participants were not differentially likely to drop-out of the study between conditions.

#### Criterion 2 - Missing data

We examined missing data among those participants that finished the study. Participants in the *all-at-once* condition were less likely to complete all 17 of the survey items (68.0% completed all) than those in the *one-at-a-time* condition (79.2% completed all items with something other than “decline to answer”). Simultaneously, participants in the *all-at-once* condition more likely to skip the survey completely (18.1% answered none of the items) than those in the *one-at-a-time* condition (12.3% answered none of the items). Of the 17-items, an average of 2.23 items (*SD* = 5.39) were missing data for the *all-at-once* condition compared to an average of 1.09 items (*SD* = 3.84) for the *one-at-a-time* condition (*t*[10735] = 8.91, *p*<.0001, *d* = .17).

#### Criterion 3 - Internal consistency

Substantially more participants completed the survey items in the *one-at-a-time* condition than the *all-at-once* condition. However, completing the items is not necessarily a good thing. Participants that would have otherwise skipped the entire survey might be less likely to pay attention or answer honestly while responding in the *all-at-once* condition. This would get more data, but at the cost of increasing measurement error and damaging the psychometrics of the survey measures. Correlations among inter-related items can be underestimated due to measurement error and other noise produced by procedures. They can also be overestimated by procedural factors that lead participants to have a consistent pattern of responding that is not a consequence of the measured construct. So, internal consistency is not a perfect criterion. However, if the measures do not differ in their tendency to elicit systematic responding, then higher internal consistency suggests better measurement properties of the procedure.

Internal consistency of the SDO was nearly identical between the *all-at-once* (alpha = .880) and *one-at-a-time* formats (alpha = .878; see also [4]). This is particularly notable considering that so many more participants were removed from the *all-at-once* condition (remaining *N* = 3560) than *one-at-a-time* condition (*N* = 4356) because of missing data on one or more items. These alpha estimates were very similar when deleting the fastest 5% of respondents who are most likely to be contributing systematic, but construct-irrelevant variance (alphas for both formats were .875). Overall, this suggests that survey procedure has no direct impact on internal consistency despite eliciting survey answers from substantially more participants than would have otherwise responded.

#### Criterion 4 - Validity correlations with criterion variables

A stronger criterion than internal consistency is the relation of a measure with variables with which it is known to be related. Criterion validity scores should be higher for the better procedure. SDO has a variety of known correlates that served as variables for criterion validity. Correlations between the two formats were nearly identical for each of the criterion variables: political orientation (.241,.246), gender (.241,.251), age (.238,.227), preference for light-skinned people over dark-skinned people (.321,.309), warmth toward dark-skinned people (−.257, −.258; *all-at-once* presented first, *one-at-a-time* presented second).

We also measured participants’ implicit evaluations using the IAT. On average, replicating prior research [9], participants showed an implicit preference favoring light-skinned people over dark-skinned people (*M* = 0.34, *SD* = .41). As expected, IAT scores were positively correlated with SDO and self-reported attitudes and these relationships did not differ significantly by survey format. The IAT was weakly positively correlated with SDO in both the *all-at-once* (*r* = .10) and *one-at-a-time* (*r* = .12) conditions. Likewise, the IAT was positively related to self-reported preferences for light-skinned people over dark-skinned people (*r* = .21 for both formats), and negatively related to reported warmth toward dark-skinned people (*r*’s =  −.12, −.14 for *all-at-once* and *one-at-a-time* respectively), and not related to reported warmth toward light-skinned people (*r*’s <.02).

In summary, the response format manipulation had no effect on the criterion validity of the measures. The fact that *one-at-a-time* substantially decreased the number of participants skipping the survey this result suggests that there is a significant gain in power – more data from more participants with no loss of reliability or validity – by using the *one-at-a-time* procedure.

#### Criterion 5 - Time required to complete the survey

All else being equal, if measures can be administered more quickly, that time efficiency respects the participant and also may give the researcher the opportunity to collect more data. For participants that completed all 17 items, participants completing the *all-at-once* condition were very slightly faster (*M* = 149 seconds, *SD* = 92) than those completing the *one-at-a-time* condition (*M* = 154, *SD* = 100; *t*[7914] = 2.12, *p* = .03, *d* = .05). It is possible that the fact that the other measures in the study were in the *all-at-once* format produced an adjustment lag to the novel *one-at-a-time* format. In Experiment 2, we converted all surveys in the study to one format or the other, and with the longer survey length (28 or 58 items instead of 17) we found that the *one-at-a-time* condition performed better on this criterion.

## Experiment 2

In Experiment 1, we observed clear evidence that the *one-at-a-time* format performed better on our criteria by eliciting more data without any loss to internal consistency or measurement validity. In Experiment 2, we sought to replicate the findings from Experiment 1 and expand the evaluation of the *one-at-a-time* format. Instead of manipulating the format of just one survey, in Experiment 2, we manipulated the presentation format of all surveys in the study. The benefits or costs of the survey formats should be more evident if when all survey measures in the study use the format. Also, we manipulated the total number of items in the event that the relative advantages differ by the number of items completed. A random half of the participants completed the Moral Foundations Questionnaire (*MFQ*, 30-items) [15] in addition to the demographics survey (13-items) and the attitudes survey (15-items). Note that “all at once” means that all of the items of a single survey were presented on one web page. So, participants that received the MFQ received three separate pages of 30, 13, and 15 items, on for each of the surveys. In the *one-at-a-time* condition, those surveys were likewise presented as distinct, with items appearing separately.

### Methods

#### Participants

Visitors to Project Implicit (https://implicit.harvard.edu/) selected to participate in the “weight” task from a group of about a dozen studies. 38168 sessions were initiated between April 8, 2010 and June 8, 2010. The participants of 26773 of those sessions remained in the study all the way through the debriefing (70%). Of participants completing demographic information, 68% were female, the average age was 28 (*SD* = 12.5), and 73% were White, 4% East Asian, 3% South Asian, 7% Black, 5% multiracial, and the rest other or unknown.

#### Materials. *Moral Foundations Questionnaire (MFQ) [15]*

The 30-item Moral Foundations Questionnaire is comprised of five subscales measuring the extent to which participants believe that five different concerns are relevant for moral judgment - harm, fairness, in-group, authority, and purity. Graham, Haidt, and Nosek (2009) [16], for example, found that political liberals tend to be primarily concerned about violations of harm and fairness in moral judgment and much less so of other three; political conservatives, on the other hand, were slightly less concerned about harm and fairness violations and more concerned about in-group, authority, and purity than were liberals. Also, Graham et al. (2011) [15] observed that women expressed stronger moral concerns about harm, fairness and purity than did men, whereas men expressed slightly stronger concerns about authority. As such, the MFQ offers five subscales for testing internal consistency between survey procedure formats. Also, as the MFQ subscales have known relations with political ideology and gender, we used these variables to test criterion validity differences between survey procedures.

***Attitude survey.*** An attitude survey of 15-items measured people’s evaluations and beliefs about weight and people who are fat or thin. Three items of this survey are used in the present analysis: [1] rating of preference for fat or thin people on a 7-point scale from strongly prefer fat people to strongly prefer thin people, [2] rating feelings of warmth for fat people on an 11-point scale from 1 = extremely cold to 11 = extremely warm, and [3] rating feelings of warm for thin people using the same scale.

***Demographics.*** A demographics survey of 13-items measured a variety of characteristics about participants’ social identities. Two items are used for survey format evaluation: [1] participant gender (1 = female, 2 = male), and [2] social politics (1 = strongly liberal, 4 = moderate, 7 = strongly conservative).

***Implicit Association Test (IAT).*** The IAT measured association strengths between faces of thin and fat people, and words with pleasant and unpleasant meaning. It followed recommended procedures and design [9],[13]. An additional experimental manipulation of the stimulus items used in the task had no effect on performance of the IAT and did not interact with the variables reported here. IAT scores were calculated with the *D* algorithm [14] with positive values indicating an implicit preference for thin people compared to fat people, and negative values indicating an implicit preference for fat people compared to thin people.

#### Ethics statement

The University of Virginia Institutional Review Board for the Social and Behavioral Sciences approved this research and informed consent process (#2002–0232). Participants were given written informed consent prior to participation, and received a written debriefing at the end of the study session.

#### Procedure

After selecting to complete the weight task, participants initiated a session and encountered an introductory page briefly describing the study procedure and informing participants that they would receive feedback on their task performance at the end. Then, participants completed the MFQ, weight IAT, self-report measures, and demographics measures in a randomized order. The 30-item MFQ, 13-item self-reported attitude survey, and 15-item demographics survey were presented in the same format - either the *all-at-once* format or with the *one-at-a-time* procedure selected at random between participants. Half of the participants were randomly selected not to receive the MFQ, shortening the session length by about 5 minutes on average. After completing all measures, participants were given feedback on their IAT performance and debriefed.

### Results

#### Criterion 1– Attrition

Participants were randomly assigned to complete study materials including the 30-item MFQ or not. Unsurprisingly, inclusion of the additional questionnaire reduced the proportion of participants that finished the study (72.2% without the MFQ, 68.1% with the MFQ). More critically, regardless of whether the MFQ was included or not, there was no difference in participant attrition whether the surveys were presented in *all-at-once* or *one-at-a-time* formats. Without the MFQ, 71.8% of participants in the *all-at-once* condition finished the study compared with 72.7% in the *one-at-a-time* condition. With the MFQ, 67.8% of participants in the *all-at-once* condition finished the study compared with 68.3% in the *one-at-a-time* condition. The slight favoring of the *one-at-a-time* condition was not statistically reliable (*p* = .48) suggesting that presentation format has no effect on likelihood of participants dropping out of the study.

**Table 1:** Alpha internal consistency coefficients for the five subscales of the Moral Foundations Questionnaire by survey format condition.

	All-at-once	One-at-a-time
Total N	5655	6047
MFQ-Harm	0.650	0.660
MFQ-Fairness	0.581	0.622
MFQ-Ingroup	0.679	0.721
MFQ-Authority	0.686	0.715
MFQ-Purity	0.801	0.807
Average	0.679	0.705

Note: Samples include only participants that completed all MFQ items. A bootstrap comparison of alphas with 5000 runs each demonstrated that all five subscales had higher reliability in the one-at-a-time compared to all-at-once conditions (*p*’s <.001).

**Table 2:** Zero-order correlations with criterion validity variables for the five subscales of the Moral Foundations Questionnaire by survey format condition.

	Social Politics		Participant Gender	
	All-at-once	One-at-a-time	All-at-once	One-at-a-time
Total N	5099	5507	5196	5662
MFQ-Harm	−0.127	−0.104	−0.322	−0.297
MFQ-Fairness	−0.160	−0.171	−0.162	−0.127
MFQ-Ingroup	0.344	0.314	0.027	0.020
MFQ-Authority	0.392	0.365	−0.019	−0.062
MFQ-Purity	0.486	0.444	−0.099	−0.166
Average (absolute values)	0.302	0.280	0.126	0.134

Notes: Gender (Male = 1, Female = 2), Social politics (1 = strongly liberal, 7 = strongly conservative). Samples include only participants that completed all MFQ items. All correlations >.05 were significant *p*<.0001. Testing significant differences in correlations between conditions showed only 3 of 10 tests being significantly different: politics effect for purity subscale being stronger for *all-at-once* versus *one-at-a-time* (*p* = .006), and gender effect for authority (*p* = .025) and purity (*p*<.001) subscales being stronger for *one-at-a-time* than *all-at-once*.

#### Criterion 2 - Missing data

Better procedures should elicit more responding from participants. We evaluated the extent of missing data considering only those participants that finished the study either without the MFQ (*N* = 13785) or with it (*N* = 12988). Without the MFQ, there were 28 total survey items between the demographics and attitude survey. There was almost twice as much missing data in the *all-at-once* condition (*M* = 3.87, *SD* = 8.47, 13.8% of total data missing) than in the *one-at-a-time* condition (*M* = 2.02, *SD* = 5.62, 7.2% total data missing; *t*[12986] = 15.48, *p*<.0001, *d* = .27). With the MFQ, there were 58 total survey items. There was even more than twice as much missing data in the *all-at-once* condition (M = 8.23, SD = 18.32, 14.2% of total data missing) than in the *one-at-a-time* condition (*M* = 4.04, *SD* = 11.93, 7.0% of total data missing; *t*[13783] = 15.11, *p*<.0001, *d* = .26).

**Table 3:** Zero-order correlations among weight attitude measures separately for *all-at-once* (below diagonal) and *one-at-a-time* (above diagonal) conditions.

	IAT	Explicit preference	Warmth toward thin people	Warmth toward fat people
Total N	26906	23746	24215	24145
IAT	–	0.181	0.058	−0.117
Explicit preference	0.166	–	0.248	−0.353
Warmth toward thin people	0.056	0.249	–	0.363
Warmth toward fat people	−0.102	−0.367	0.347	–

Notes: Higher values for the IAT and explicit preference indicate more liking of thin people relative to fat people. Higher values on warmth measures indicate greater feelings of warmth. All correlations were significant with *p*<.0001.

For each of the three surveys, the *all-at-once* condition had more participants skip the entire survey and fewer participants complete the entire survey than the *one-at-a-time* condition. For the MFQ, 12.4% of participants skipped the entire survey in the *all-at-once* condition compared to 5.0% in the *one-at-a-time* condition; at the same time, 82.0% of participants completed every item in the *all-at-once* condition compared to 86.8% in the *one-at-a-time* condition. Similar results were observed for the demographics survey (10.4% skipped all and 77.5% completed all in *all-at-once*, 2.1% skipped all and 85.9% completed all in *one-at-a-time*) and the attitude survey (11.5% skipped all and 81.1% completed all in *all-at-once*, 3.7% skipped all and 92.0% completed all in *one-at-a-time*). One demographics item, field of study, was only relevant for a subset of respondents so missing it was not counted as a skip for these percentages. Also, the percentages here are for the MFQ-present condition, they are nearly identical when the MFQ was not present.

#### Criterion 3 - Internal consistency

Table 1 presents internal consistency estimates for each of the five MFQ subscales separately for the *all-at-once* and *one-at-a-time* conditions for participants that completed all survey items. The *one-at-a-time* condition consistently elicited slightly higher internal consistency (average alpha = .705) than the *all-at-once* condition (average alpha = .679). A bootstrap simulation compared the differences between conditions with 5000 runs each. [17] The results showed that the *one-at-a-time* condition elicited significant stronger reliability than *all-at-once* for all five subscales (all *p*’s <.001). Also, these alpha estimates were very similar for all five subscales when deleting the fastest 5% of respondents who are most likely to be contributing systematic, but construct-irrelevant variance. This suggests that the *one-at-a-time* procedure elicits slightly better responding from participants while it simultaneously reforms a significant number of participants that would have otherwise skipped completing the items at all.

#### Criterion 4 - Validity correlations with criterion variables

All of the MFQ subscales are known to be related with social politics (liberal-conservative), and most are related to gender. As such, the better procedure should elicit higher correlations between the MFQ subscales and these criterion variables on average. Table 2 presents zero-order correlations between each of the five subscales and the two criterion variables - social politics and participant gender - separated by survey format condition. Criterion validity relations replicated prior findings and the strength of the relations were very similar across format conditions. [15]–[16] Overall, it appears that the *all-at-once* condition performed slightly better than the *one-at-a-time* condition for the social politics criterion variable, and vice versa for the gender variable. However, these differences are very weak (just 3 of 10 correlation comparisons were significant with extremely high power). The condition difference for MFQ-Purity for social politics (*p* = .006), for example, corresponds to an effect size *d* of .04.

**Table 4:** Time to complete study materials (in seconds) for each survey format separately for sessions that included the MFQ or did not.

	with MFQ		without MFQ	
	All-at-once	One-at-a-time	All-at-once	One-at-a-time
Total N	1654	1685	1772	1922
MFQ	232 (107)	221 (99)	N/A	N/A
Attitude Survey	129 (54)	121 (56)	129 (58)	124 (62)
Demographics Survey	104 (61)	98 (50)	103 (61)	101 (61)
Total Time	912 (283)	866 (284)	674 (242)	650 (246)

Notes: Total time to complete the study included instructions and the IAT. *SD*s in parentheses. Sample includes only participants that finished the study and completed all items in all surveys.

A final criterion validity test compared implicit and self-reported attitudes toward fat and thin people. On average, participants reported preferring thin people to fat people (*M* = 1.01, *SD* = 1.08), and reported warmer feelings for thin people (*M* = 7.85, *SD* = 1.88) than fat people (*M* = 7.08, *SD* = 2.17). Likewise, with the IAT, participants showed an implicit preference for thin people compared to fat people (*M* = 0.45, *SD* = 0.42). Correlations among these four variables are shown in Table 3. All correlations were in the expected directions with more positivity toward thin people on one measure being associated with more positivity toward thin people on the other measures, and likewise for fat people. After taking the absolute value of the two expected (and observed) negative correlations, the average correlation for the *all-at-once* condition was 0.215 and for the *one-at-a-time* condition was 0.220. The difference between conditions was trivial.

Across criterion validity tests, survey formats did not elicit differentially valid measures. This is notable considering that the *one-at-a-time* condition had much less missing data.

#### Criterion 5 - Time required to complete the study and surveys

We compared the time required to complete the study and surveys among participants that (a) finished the study, (b) filled out all of the items, and (c) after removing excessively long study completion times (>3 std’s above the mean completion time) suggesting that the person paused during the study for long periods of time. Table 4 presents average completion times for the *all-at-once* and *one-at-a-time* conditions separately for sessions that included the MFQ and sessions that did not. Participants completed the study faster in the *one-at-a-time* condition whether the MFQ was included (*one-at-a-time M* = 866; *all-at-once M* = 912) or not (*one-at-a-time M* = 650; *all-at-once M* = 674). For every survey, the *one-at-a-time* format cut down task completion time by at least 2 seconds and, in the case of the MFQ, 11 seconds for the 30-item measure. These findings are similar to one study [1], which reported shorter completion times in their paging format. In contrast, several others report the opposite finding [4]–[7]. It is perhaps most likely that differences between the specific surveys, as well as inconsistent use of a strict *one-at-a-time* format used across the various studies may have impacted the completion time effects that are reported. For example, if the respondent’s machine communicated with the server in-between every question (the standard using HTML format), then the time required for *one-at-a-time* would be highly dependent on the speed of the internet connection and the server’s responsivity. Our *one-at-a-time* format communicated with the server just once, at the end of the survey.

## Discussion

In two experiments, for five distinct criteria, presenting survey items *one-at-a-time* was either the same or better than presenting survey items *all-at-once* on a single web page. In the *one-at-a-time* format, volunteer participants were no more likely to drop-out of the study (Criterion 1), and were much more likely to provide answers for the survey items (Criterion 2). Rehabilitating participants who otherwise would not have provided survey responses with the *one-at-a-time* format did not damage internal consistency of the measures (Criterion 3) nor did it negatively affect criterion validity (Criterion 4). Finally, the *one-at-a-time* format was more efficient with participants completing it more quickly than the *all-at-once* format for Experiment 2 that include more items in total (Criterion 5). In short, the *one-at-a-time* format results in less missing data with a shorter presentation time, and ultimately more power to detect relations among variables.

These benefits suggest that the *one-at-a-time* format will be quite useful, especially in circumstances that the participants are volunteers who may have minimal incentives to remain in the study unless they are enjoying or finding other value in responding. Previous findings in the literature have been non-committal in terms of stating that one format produces qualitatively better results than another in an attempt to avoid the conclusion that a single format should be used in all cases [4]–[5]. While we recognize that a single format is not likely to be the best solution in all cases, the current studies provide convincing evidence that the *one-at-a-time* format is a wiser methodological choice for many web-based surveys. While these studies had enormous samples making power a non-issue, a clear benefit of the results is that studies with smaller samples are likely to benefit the most from the *one-at-a-time* format because of increased power. The difference in survey procedure reduced missing data by about 50% in both studies. For studies in which every item and every response counts, that improvement can be the difference between an appropriate powered, significant result and an underpowered, non-interpretable result.

### Limitations

There are an infinite variety of alternative survey procedures to the *all-at-once* and *one-at-a-time* varieties described here. There most certainly exist cases in which presenting more than one item at a time will improve measurement - for example, if item responses are interdependent and it would be useful for respondents to be able to see previous responses when making subsequent responses. Likewise, the context of participation is likely to play an important role in the comparative effectiveness of different survey procedures. For example, as the present participants were volunteers, attrition was a substantial concern and criterion for evaluating procedure quality. However, attrition is much less of an issue if participants are unlikely to drop-out of the study, such as when participants visit the laboratory or are compensated only if they complete all study materials. In short, the generality of these conclusions depends on whether the data collection circumstances elicit similar issues for the evaluation criteria applied here.

### Conclusion

Experimental evaluation of survey procedural formats is not the kind of research that tends to drive dinner conversation – even among nerds. Even so, the procedures of data collection are vital to the efficient, reliable and valid collection of survey data that is the substance of most behavioral research. The present results provide evidence of a clear information gain by using a *one-at-a-time* presentation format for surveys, at least among web-based volunteer participants. Adopting this practice will increase the likelihood that researchers will obtain results that can provide scintillating anecdotes for the dinner party, and impactful evidence for building scientific knowledge.
